# Assessment of Pain, Vital Parameters and Oxidative Stress Markers in Dogs After Celiotomy and Three‐Port Laparoscopic Ovariectomy

**DOI:** 10.1002/vms3.70683

**Published:** 2025-11-12

**Authors:** Reza Naghavi, Hossein Kazemi Mehrjerdi, Mohammad Heidarpour

**Affiliations:** ^1^ Department of Clinical Sciences Faculty of Veterinary Medicine Ferdowsi University of Mashhad Mashhad Iran

**Keywords:** dogs, laparoscopic ovariectomy, midline ovariectomy, oxidative stress

## Abstract

The present study evaluated the surgical stress response in dogs undergoing ovariectomy (OVE) using celiotomy and laparoscopy techniques. Twelve clinically healthy, intact bitches, with an average weight of 20–25 kg, were randomly and equally divided into two groups: celiotomy ovariectomy (COVE) and laparoscopy with a three‐portal midline technique (laparoscopic ovariectomy [LapOVE]). A clinical assessment was conducted, and haematological parameters and oxidative stress biomarkers were measured at baseline and 1, 24 and 168 h after extubation. Pain scoring was performed using the University of Melbourne Pain Scale at 2, 4, 8 and 12 h postoperatively.

The average heart rate, respiratory rate, rectal temperature, SpO_2_ saturation and EtCO_2_ values showed no significant differences within each group or when comparing the two groups. There was no significant difference in the pain evaluation between the groups. At 168 h postoperatively, both malondialdehyde (MDA) and total antioxidant capacity (TAC) were significantly higher in the COVE group compared with LapOVE (MDA: 6.1 ± 0.844 vs. 4.1 ± 0.848; *p* < 0.05; TAC: 0.294 ± 0.068 vs. 0.246 ± 0.023; *p* < 0.05). The COVE group exhibited significantly higher white blood cell (WBC) and neutrophil counts, and lower eosinophil counts, than the LapOVE group (all *p* < 0.05).

The findings from this research indicated that both surgical methods were safe and yielded similar pain and physiological results. The COVE group demonstrated significantly increased oxidative and inflammatory responses 1 week after surgery, suggesting a higher level of surgical stress than the LapOVE group. When available, laparoscopic OVE should be preferred as a minimally invasive option to reduce postoperative inflammation and oxidative stress.

## Graphical abstract

This research compares laparoscopic and open ovariectomy in bitches, focusing on surgical stress, pain, oxidative stress and immune response. Laparoscopy resulted in lower postoperative inflammation and oxidative injury. Despite similar pain scores, the minimally invasive approach demonstrated clinical benefits, endorsing its use where equipment and skills are available.

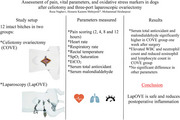

## 1 Introduction

Ovariectomy (OVE) is a widely performed surgical procedure in dogs (Schwarzkopf et al. 2015; Watts 2018). It is typically carried out for population control, preventing diseases such as pyometra and mammary tumours and behavioural modification (Kumar et al. 2023). Traditionally, OVE in dogs has been performed via a celiotomy approach, which involves a larger incision to access the abdominal cavity and ovaries (Howe 2006). However, with advances in surgical equipment, laparoscopic ovariectomy (LapOVE) has gained popularity as a minimally invasive alternative (Huhn 2016). LapOVE utilizes small incisions and a camera‐guided system to remove the ovaries, offering potential advantages over conventional open ovariectomy (celiotomy ovariectomy [COVE]), including reduced postoperative pain and stress, shorter recovery times and faster return to normal activity (Serin et al. 2008). However, despite its benefit, the insufflation of CO_2_ during laparoscopy can cause peritoneal acidosis and oxidative stress through ischemia–reperfusion mechanisms (Safran and Orlando 1994). These dual effects of laparoscopy necessitate a detailed investigation into oxidative markers such as malondialdehyde (MDA) and total antioxidant status (TAS) to better understand the procedure's overall oxidative impact.

As shown in dogs, oxidative stress and immune responses are significant factors in postoperative recovery (Devitt et al. 2005; Dalmolin et al. 2024). Oxidative stress, which results from a disparity between reactive oxygen species (ROS) and antioxidant defences, has the potential to lead to cellular damage and hinder recovery (Tomsič and Nemec Svete 2022; Azizi et al. 2025; Nazari et al. 2025). In major surgical procedures, it manifests acutely, primarily during ischemia–reperfusion injury, as evidenced during laparoscopy, mechanical stress and the inflammatory response to tissue damage (Dalmolin et al. 2024). The selection of intraoperative methods, procedures and materials frequently hinges on attempts to minimize trauma and decrease surgical time (Watts 2018).

MDA results from lipid peroxidation, and its concentrations are frequently utilized as indicators of oxidative damage (Azizi et al. 2025; Costa et al. 2024). Elevated MDA levels signify intensified oxidative stress, which may impede tissue regeneration and result in postoperative complications, such as delayed wound healing and infections (Dissemond et al. 2002; Nemec Svete et al. 2021). Conversely, TAS indicates the body's ability to counteract oxidative stress by assessing the total antioxidant capacity (TAC). A decrease in TAS postoperatively may suggest that the body's antioxidant reserves have depleted during surgery (Tomsič and Nemec Svete 2022).

The postoperative period is crucial in any surgical intervention, with pain and inflammation significantly influencing healing. Pain is frequently more severe and enduring after open surgery because of larger incisions, increased tissue dissection and more extensive interior exposure (Devitt et al. 2005; Culp et al. 2009; Hancock et al. 2005). Laparoscopy is commonly linked to decreased postoperative discomfort; nevertheless, the visceral pain induced by CO_2_ pneumoperitoneum poses a distinct difficulty (del Romero et al. 2020). Inadequate pain control can result in behavioural changes, extended recovery and diminished overall results; therefore, effective postoperative pain management is essential.

Haematological changes are crucial for understanding the physiological response to surgery. Postoperative changes in white blood cell (WBC) counts, including differential counts of neutrophils, eosinophils, lymphocytes and monocytes, provide critical insights into immune system activation (Rubio et al. 2022). It is crucial to investigate the differences in immunological responses between LapOVE and COVE, as they may influence postoperative management and recovery times (Rubio et al. 2022).

This research compared LapOVE with COVE in canines, emphasizing postoperative pain, oxidative stress and haematological changes. This study aimed to clarify the comparative advantages and disadvantages of laparoscopic and open OVE, thereby improving the welfare and recovery of canine patients.

## 2 Materials and Methods

### 2.1 Animals

All procedures involving the handling of animals and methodologies were sanctioned by the ethics committee at Ferdowsi University of Mashhad. Twelve healthy, mixed‐breed bitches, aged approximately 6–24 months and weighing 20–25 kg, were initially included. Animals were obtained from a local animal shelter, and permission was acquired from the legal guardians of all animals mentioned in this protocol for all procedures performed.

Each dog was kept in its cage within a 12‐h light and 12‐h dark cycle and given a standard commercial diet (300 g/dog/day; Nutripet; Behintash Co., Karaj, Iran). Access to water was provided to the dogs ad libitum. During the initial 3 weeks of acclimatization, the dogs received an anthelmintic tablet (fenbendazole, 150 mg; pyrantel embonate, 144 mg; praziquantel, 50 mg; Caniverm, 0.7 mg/10 kg, orally). The overall health of the animals was evaluated through clinical examinations followed by laboratory analyses, and the pregnancy status of the dogs was determined using abdominal ultrasonography. The patients were randomly allocated into two groups based on the type of surgical technique: COVE (*n* = 6) or LapOVE (*n* = 6).

### 2.2 Anaesthesia and Analgesia

The bitches underwent a 12‐h fasting period prior to the surgery while having unrestricted access to water. Preanaesthetic medications, including 0.05 mg/kg acepromazine (Neurotranq 1%, Alfasan Co., the Netherlands) and 0.5 mg/kg morphine (intramuscularly [IM]; Darou Pakhsh Co., Iran), were given IM. Following sedation, a 20‐ga catheter was placed into the cephalic vein, and Ringer's lactate solution (5 mL/kg/h) was given throughout the surgery. A combination of 0.25 mg/kg diazepam (Zepadic, Caspian Co, Iran) and 6 mg/kg ketamine (Ketaset 10%, Alfasan Co., the Netherlands) was used to induce general anaesthesia. The animals were intubated with an endotracheal tube and were breathing spontaneously. General anaesthesia was maintained with isoflurane gas (2% end‐tidal concentration) (Terrel, USA) and 100% oxygen. The level of anaesthesia was evaluated on the basis of the jaw's muscular tone, the eyeball's position, the pupil's size and the dog's reaction to surgical procedures. The dogs were administered 0.2 mg/kg of meloxicam (Rooyan Co., Iran) via subcutaneous injection and 22 mg/kg of cefazolin (AFAZOLE, Iran) intravenously as prophylaxis 30 min before the surgery. Meloxicam was administered again 3 days after the surgical procedure (Fernández‐Martín et al. 2022).

### 2.3 Surgical Procedures

All procedures commenced with dogs in dorsal recumbency. The identical veterinary surgical team conducted all the operations in both groups. LapOVE was performed utilizing a three‐portal approach with linear access to the abdomen (Azizi et al. 2025; Hasson 1999; Kumari et al. 2022). A Veress needle was inserted 1 cm caudal to the umbilicus at midline, and carbon dioxide (CO_2_) was insufflated (Olympus UHI‐2, Olympus, Japan) at a constant rate of 1 L/min until the intraabdominal pressure (IAP) reached 8–10 mmHg. The Veress needle was removed, and in the same punction, an 11 mm trocar was inserted in the region for a 10 mm 0° rigid endoscope (Olympus CLO‐S40, Olympus, Japan) attached to a camera placement (sub‐umbilical port). Instrument portals (5 mm) with optic visualization were placed 3–5 cm cranial (between the xiphoid cartilage and umbilicus) and caudal (between the pubis and umbilicus) to the first port.

For identification of left ovary, the bitch was manually rotated into right lateral oblique recumbency and tilted to the right side in a 10° Trendelenburg position (head down). The ovary was located and suspended with Kelly forceps, and then the ovarian pedicle and fallopian tube were carefully cauterized and transected with 5 mm bipolar electrocoagulation forceps. Once the ovary was cut free, haemostasis of the ovarian pedicles was verified. The bitch was tilted to the left side, and the same manoeuvre was carried out for the right ovary. Upon confirmation of the absence of bleeding in the abdominal cavity, both resected ovaries were removed through the mid‐cannula, and the pneumoperitoneum was purged. Portal sites were closed with separate sutures in the abdominal fascia, subcutaneous tissue and skin.

In the celiotomy group, the incision was made on the midline, 1/3 of the distance between the umbilicus and pubis. Following a ventral median celiotomy, the ovarian pedicles were secured with forceps, ligated twice with sutures and cut. The fallopian tube and the proper ovarian ligament were secured with sutures, and the ovary was excised. The external rectus fascia and subcutaneous tissue were closed in two separate layers with a simple continuous pattern using 2–0 Polyglactin 910 (Vicryl, Ethicon). The skin was sutured with 3–0 nylon in an interrupted cruciate suture pattern.

### 2.4 Postoperative and Blood Sampling

The surgical time from the moment of skin incision or Veress needle placement to the time of last suture placement was recorded for each dog. Assessment of heart rate (HR; beats/min), respiratory rate (RR; breaths/min), rectal temperature (RT; °C), peripheral capillary oxygen saturation (SpO_2_ saturation) and end‐tidal CO_2_ (EtCO_2_) levels was performed for each dog using a multiparametric monitor (cardio set, ARAD P10, SaIran Medical Industrial, Iran) during anaesthesia and surgery. The parameters were recorded manually every minute throughout the surgical procedure, whereas the statistical analysis of the data was conducted using values recorded at four distinct times: before CO_2_ insufflation at the beginning of surgery, after the first OVE, after the second OVE and at the time of skin suture.

Pain levels were evaluated at 2, 4, 8 and 12 h post‐surgery utilizing the University of Melbourne Pain Scale (UMPS), which is a validated multidimensional instrument designed to assess postoperative pain in dogs. This scale consists of six categories: physiological parameters (such as HR and RR), reaction to palpation, activity level, mental condition, posture and vocal responses. Each category receives an individual score, and the overall pain score can range from 0 to 27 (Firth and Haldane 1999). A cumulative score exceeding 6 indicated moderate‐to‐severe pain, prompting the administration of rescue analgesia (morphine, 0.3 mg/kg IM).

Blood samples were obtained through the jugular vein before the administration of any anaesthetic or premedication drugs (at baseline) and at 1, 24 and 168 h following extubation. Blood was sequentially transferred into two Vacutainer tubes, one containing an ethylenediaminetetraacetic acid (EDTA) tube and the other a plain tube. The serum was centrifuged at 3000 rpm for 10 min, stored in 2 mL tubes and preserved at −80°C to assess MDA and TAC laboratory parameters.

For the WBC count, 0.5 mL of blood was drawn through jugular venipuncture, placed in EDTA tubes (Labor 0.5 mL) simultaneously and processed without delay. An additional aliquot (5 mL) was collected, put into a serum separator tube and centrifuged at 3000 × *g* for 10 min. Each serum sample was divided into aliquots (400 µL per Eppendorf‐type tube) and kept at −80°C until further analysis.

### 2.5 Laboratory Measurements

#### 2.5.1 MDA

In this study, serum MDA was assessed using the spectrophotometric technique established by Placer and colleagues (Kumari et al. 2022). The reaction mixture consisted of 0.2 mL of serum, 1.3 mL of 0.2 M Tris–0.16 M KCl buffer at pH 7.4 and 1.5 mL of thiobarbituric acid reagent. The solution was then heated in a boiling water bath for 10 min. After cooling, 3 mL of a pyridine/*n*‐butanol mixture (3:1, v/v) and 1 mL of 1 N sodium hydroxide solution were added, and the mixture was thoroughly mixed by shaking. A control without any sample was prepared simultaneously by replacing 0.2 mL of serum with distilled water. The sample's absorbance was measured at a wavelength of 548 nm. The concentration of MDA in nmol per mL of serum was calculated using the formula: MDA (nm/mL) = (V*OD548)/0.152.

#### 2.5.2 Ferric Reducing Antioxidant Power (FRAP)

The FRAP assay evaluated the TAC. This assay's basis is reducing ferric tripyridyl‐*s*‐triazine (Fe3þ‐TPTZ) complex to ferrous tripyridyl‐*s*‐triazine (Fe2þ‐TPTZ) at low pH. Fe2þ‐TPTZ generates a blue colour that can be measured at 593 nm by a spectrophotometer. Calculations were performed using a calibration curve of FeSO_4_·7H_2_O (100–1000 mM).

### 2.6 Haematological Analysis

Haematological parameters in blood samples treated with anticoagulants were analysed using an automated haematology analyser (Celltac Automated Hematology Analyzer MEK 6450, Nihon Kohden, Japan). The evaluation included haemoglobin (Hb), haematocrit (HTC), red blood cell (RBC) count, total WBC count, mean corpuscular volume (MCV), mean corpuscular haemoglobin (MCH), mean corpuscular haemoglobin concentration (MCHC), platelets (Plt) and total protein (TP). Additionally, a differential leukocyte count was conducted microscopically on the same samples for lymphocytes, monocytes, adult neutrophils, basophils and eosinophils, utilizing the Giemsa staining method on blood films through a cross‐sectional approach. Blood smears were made, air‐dried and fixed in methanol. Subsequently, the microscopic slides were placed in a diluted Giemsa solution for 30–40 min. Light microscopy (Helmut Hund GmbH, Germany) was performed after washing with tap water and allowing air drying.

### 2.7 Statistical Analysis

The data were examined using SPSS software (version 26). The Shapiro–Wilk test was employed to assess the data for normality. Age, weight and surgical time were compared between groups using an independent sample *t*‐test. For physiological, haematological and oxidative stress markers measured at multiple time points, a repeated measures ANOVA analysis was conducted. The fixed factors in the model were group (LapOVE vs. COVE), time (baseline and postoperative time points) and the group × time interaction. Mauchly's test of sphericity was used to evaluate the sphericity assumption; when violated, the Greenhouse–Geisser adjustment was used. Post hoc pairwise comparisons were performed using Dunnett's test (to compare each postoperative time point against baseline within groups), and Bonferroni correction was applied to control the familywise error rate across multiple comparisons. The Mann–Whitney test was used to compare pain scores between the groups. Data that follow a normal distribution are expressed as means ± standard deviation. Non‐normally distributed variables were represented as median and interquartile range (Q1–Q3). The significance level for all variables was considered *p* < 0.05.

## 3 Results

All dogs recovered uneventfully, and there were no surgical or postoperative complications. The sample characteristics by group are described in Table 1. Dogs aged 6–24 months (17.9 ± 1.73) and weighing 20–25 kg (22.07 ± 1.73). The total surgical time was 19.21–23.40 min (21.12 ± 1.31), and the total anaesthetic time was 39.21–42.80 min (40.57 ± 1.00). The total anaesthetic time was 39.21–40.70 min (39.98 ± 0.61) in the COVE group and 39.80–42.80 min (41.16 ± 0.98) in the LapOVE group. On the basis of the Melbourne scale, no significant difference existed between groups; no patient scored greater than 6 (0–27) (Table 2). Consequently, no patient needs extra pain relief medication. The mean HR, RR, RT, SpO_2_ saturation and EtCO_2_ did not differ between the four points in each group or between the two groups (Table 3).

**TABLE 1:** Mean ± standard deviation value of weight, age and procedural times in both groups during ovariectomy surgery via ventral celiotomy and laparoscopy technique.

	COVE	LapOVE	*p* value
Weight (kg)	22.36 ± 1.96	21.78 ± 1.59	0.565
Age (months)	17.36 ± 4.78	18.43 ± 6.36	0.531
Surgery time (min)	20.16 ± 0.82	22.13 ± 0.89	0.711
Anaesthesia time (min)	39.98 ± 0.61	41.16 ± 0.98	0.674

Abbreviations: COVE, celiotomy ovariectomy; LapOVE, laparoscopic ovariectomy.

**TABLE 2:** Cumulative pain score in both groups during ovariectomy surgery via ventral celiotomy and laparoscopy techniques.

	2 h	4 h	8 h	12 h
Midline ovariectomy	1.5 (1–2.25)	3 (2–4.25)	2.5 (1.75–4.25)	3 (1.75–3.25)
Laparoscopy ovariectomy	1.5 (1–2)	2.5 (1–3.25)	2 (1.75–3.5)	2 (1–3.25)
*p* value	0.78	0.28	0.67	0.40

**TABLE 3:** Physiological parameters (mean ± SD) evaluated in 12 dogs submitted to celiotomy ovariectomy (COVE) and laparoscopic ovariectomy (LapOVE).

		Time			
Variable	Group	Before surgery	First ovariectomy	Second ovariectomy	Skin suture
HR	COVE	107.83 ± 10.14	111.33 ± 8.26	112.00 ± 8.02	111.50 ± 7.14
	LapOVE	109.33 ± 7.63	111.83 ± 6.24	112.33 ± 6.06	112.83 ± 7.6
RR	COVE	13.50 ± 1.76	13.83 ± 4.57	13.66 ± 3.20	13.50 ± 3.08
	LapOVE	13.66 ± 1.03	14.00 ± 1.26	13.83 ± 2.13	13.66 ± 3.07
RT	COVE	38.08 ± 0.19	38.03 ± 0.18	38.01 ± 0.13	38.01 ± 0.18
	LapOVE	38.15 ± 0.13	38.06 ± 0.12	38.00 ± 0.16	38.00 ± 0.20
SpO_2_	COVE	98.10 ± 0.60	97.56 ± 0.63	98.00 ± 0.70	98.08 ± 0.49
	LapOVE	97.85 ± 0.83	97.83 ± 0.51	98.16 ± 0.83	97.50 ± 0.54
EtCO_2_	COVE	40.78 ± 1.95	40.95 ± 1.84	40.15 ± 2.04	39.56 ± 4.95
	LapOVE	39.58 ± 1.81	39.56 ± 1.86	39.85 ± 2.38	40.41 ± 1.98

Abbreviations: EtCO_2_, end‐tidal CO_2_; HR, heart rate; RR, respiratory rate; RT, rectal temperature; SpO_2_, peripheral capillary oxygen saturation.

### 3.1 Changes of Oxidative Stress Markers in the LapOVE and COVE Groups

**FIGURE 1:**
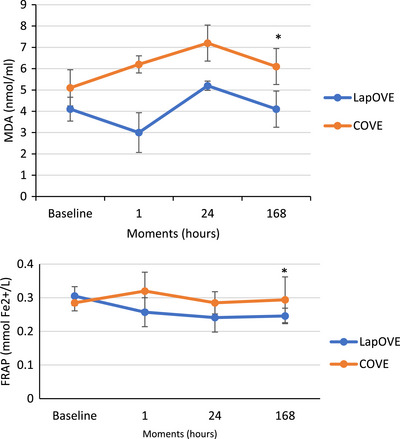
Line graph of (A) malondialdehyde (MDA) and (B) total antioxidant capacity (TAC). Mean values ± standard deviation of each concentration of both groups are on the vertical axis, and the time (baseline, 2, 24 and 168 h after surgery) is on the horizontal axis. Asterisks (*) mean significant differences between groups (*p* < 0.05). FRAP, ferric reducing antioxidant power.

The oxidative stress marker values (mean ± SD) for the LapOVE and COVE groups are shown in Table 4. Repeated measures ANOVA revealed that the group significantly affected FRAP and MDA (*p* < 0.05). The results of oxidative stress markers at various time points are shown in Figure 1. Although the MDA and FRAP levels were significantly higher in the COVE group than in the LapOVE group 1 week after surgery (*p* < 0.05), there were no differences among measurement moments within each group.

**TABLE 4:** Mean ± standard deviation value of malondialdehyde (MDA) and total antioxidant capacity (TAC) in both groups during ovariectomy surgery via ventral celiotomy and laparoscopy technique.

Oxidative stress markers	Groups		Significant level		
	Laparoscopy	Celiotomy	Time	Group	Time × group
MDA (nmol/mL)	4.4505 ± 1.527	6.723 ± 1.460	0.20	0.025	0.704
FRAP	0.259 ± 0.034	0.304 ± 0.045	0.15	0.036	0.301

Abbreviation: FRAP, ferric reducing antioxidant power.

### 3.2 Changes in Haematology Parameters in the LapOVE and COVE Groups

Table 5 shows haematology parameters’ values (mean ± SD) in the LapOVE and COVE groups. Repeated‐measures ANOVA revealed that time had a significant effect (*p* < 0.05) on packed cell volume (PCV), HB, RBC, MCHC, WBC, TP, lymphocyte and neutrophil values. The group had a significant effect (*p* < 0.05) on WBC, neutrophils and eosinophils. WBC and neutrophil counts were significantly higher in the COVE group than in the LapOVE group at all times. In contrast, eosinophil counts were significantly lower than in the LapOVE group 1 week after surgery. In both groups, neutrophil and WBC counts decreased 2 h after surgery compared to the baseline values, but this decrease was significant only in the laparoscopic group. There were no differences in RBC, MCV, MCH, MCHC and Plt between groups or at measurement moments within groups. Although there was no difference in the TP between groups, the LapOVE group showed an increase at 1 week (*p* < 0.05). In the COVE group, lymphocyte counts showed a significant decrease at 2 h, accompanied by reductions in Hb and eosinophil counts at both 2 and 24 h after surgery compared to baseline levels.

**TABLE 5:** Mean ± standard deviation values of some blood parameters at different time intervals in both groups during ovariectomy surgery via ventral celiotomy and laparoscopy techniques.

Parameters	COVE				LapOVE			
	Baseline	1 h	24 h	168 h	Baseline	1 h	24 h	168 h
PCV (%)	24.30 ± 8.52^A^	33.80 ± 5.65^B^	36.52 ± 5.37	38.28 ± 5.48	42.32 ± 7.12^A^	34.34 ± 6.48^B^	41.66 ± 3.20	42.42 ± 4.88
Hb (g/dL)	13.60 ± 1.98^A^	11.66 ± 2.03^B^	12.26 ± 2.18^B^	13.24 ± 2.22	15.06 ± 1.79	13.48 ± 0.87	14.58 ± 1.16	14.52 ± 1.88
RBC (×10^6^/µL)	7.33 ± 2.57	5.02 ± 0.49	5.39 ± 0.65	5.88 ± 0.55	6.47 ± 0.91	5.738 ± 0.37	6.20 ± 0.34	6.13 ± 0.74
MCV (fL)	66.18 ± 6.47	67.18 ± 6.51	67.70 ± 6.12	67.92 ± 5.61	66.32 ± 2.67	66.14 ± 3.10	66.42 ± 2.63	69.12 ± 3.76
MCH (pg)	23.36 ± 2.49	23.16 ± 2.52	22.72 ± 3.02	23.44 ± 2.52	24.28 ± 2.02	23.32 ± 1.11	24.14 ± 1.77	23.64 ± 1.32
MCHC (g/dL)	35.28 ± 1.91	34.28 ± 1.12	33.42 ± 1.48	34.42 ± 0.93	34.92 ± 1.15	34.60 ± 0.92	33.88 ± 0.83	34.22 ± 0.72
TP	6.02 ± 0.40	6.06 ± 0.45	6.98 ± 0.50	7.02 ± 0.43	6.84 ± 0.51^A^	6.28 ± 0.49	6.64 ± 0.85	7.54 ± 0.29^B^
Plt (×10^5^/µL)	3.39 ± 1.32	3.22 ± 0.81	3.33 ± 1.545	4.48 ± 1.29	2.76 ± 0.46	2.47 ± 0.50	2.816 ± 0.46	2.92 ± 0.67
WBC	14,180 ± 2543.02	9580 ± 2397.29^*^	16,140 ± 6285.93^*^	15,680 ± 3509.55^*^	8570 ± 2338.16^A^	6470 ± 1219.42^B^ ^*^	10,800 ± 3962.32^*^	11,660 ± 3142.92^*^
Eosinophil (µL)	141.8 ± 25.43^A^	81.400 ± 49.68^B^	33.20 ± 74.23^B^	133.40 ± 159.04^*^	305.6 ± 264.67	331.60 ± 258.95	282.60 ± 291.66	589.6 ± 494.13^*^
Lymphocyte (µL)	2112.6 ± 798.30^A^	1207.6 ± 166.98^B^	1917.8 ± 728.26	2817 ± 512.36	2102.8 ± 1006.37	1218.6 ± 687.62	2229.2 ± 1575.55	2671.6 ± 1035.27
Monocyte (µL)	748.20 ± 409.91^A^	507.80 ± 235.15^B^	1581.8 ± 798.94^*^	1127.2 ± 447.32	481 ± 197.82	309.60 ± 28.34	426.60 ± 384.71^*^	646.80 ± 153.77
Neutrophil (µL)	11,445 ± 2260.20	8139.4 ± 2561.41^*^	13,764 ± 6054.22^*^	12,083 ± 2864.90^*^	5453.4 ± 1710.32^A^	4340 ± 892.22^B^ ^*^	6875.8 ± 2561.24^*^	6907.6 ± 2013.08^*^

*Note*: Different capital letters in superscript indicate significant differences within the surgical group.

Abbreviations: CBC, complete blood count; COVE, celiotomy ovariectomy; Hb, haemoglobin; HCT, haematocrit; LapOVE, laparoscopy ovariectomy; MCH, mean corpuscular haemoglobin; MCHC, mean corpuscular haemoglobin concentration; MCV, mean corpuscular volume; PCV, packed cell volume; Plt, platelets; RBC, red blood cell; TP, total protein; WBC, white blood cell.

* Significant difference compared to the other surgical group's variable and time point.

## 4 Discussion

This study compared the effects of two different OVE techniques in dogs, focusing on oxidative stress, haematological parameters and pain scores. Studies have shown that various OVH techniques in dogs have advantages and disadvantages (Schwarzkopf et al. 2015; Watts 2018; Caroff et al. 2019). The incidence of complications after laparoscopic OVH and conventional celiotomy OVH was 2% and 7.5%–19%, respectively (Dalmolin et al. 2024; Caroff et al. 2019; Freeman et al. 2010).

The surgery duration depends on the surgical procedure, techniques, equipment and the surgeon's proficiency. In more extended operations, the possibility of complications is higher (Lee and Kim 2014). No matter the method used, extended durations of surgery are linked to heightened oxidative stress and inflammatory reactions caused by prolonged manipulation of tissues and exposure to anaesthesia (Schwenk et al. 2000). In our study, no problems were observed in the surgery of the LapOVE and COVE groups, and the duration of surgery did not differ between groups. Different studies have yielded inconsistent findings regarding the duration required for laparotomy and laparoscopic OVE/OVH procedures in dogs. Some studies reported shorter times (Del Romero et al. 2020; Fernández‐Martín et al. 2022; Hasson 1999), whereas others reported longer surgical times associated with laparoscopic OVE/OVH versus open OVE/OVH (Dalmolin et al. 2024; Culp et al. 2009; Espadas‐González et al. 2023; Gauthier et al. 2015).

This study observed no significant difference between the two groups regarding HR, RR, RT, SpO_2_ saturation and EtCO_2_ indices during surgery. This outcome corresponds with prior research demonstrating that both laparoscopic and open techniques preserve steady physiological characteristics throughout the operation (Hancock et al. 2005; Lee and Kim 2014; Bruschetta et al. 2024). Hancock et al. (2005) noted that there was no significant difference in RT in dogs following ovariohysterectomy performed via laparoscopy compared to median celiotomy (Hancock et al. 2005). Certain studies indicated that the laparoscopy group exhibited a lower RT than the celiotomy group at the end of surgery in cats. This observation was ascribed to the prolonged anaesthesia duration in the laparoscopic group and the administration of unheated CO_2_ into the abdominal cavity (Gauthier et al. 2015). However, although the duration of anaesthesia was longer in the LapOVH group in the study by Dalmolin et al., hypothermia was not observed. The authors suggested this was probably due to the lack of exposure of abdominal organs, low blood loss and the animals being positioned in a lateral recumbent position, which minimized body temperature changes (Dalmolin et al. 2024; Redondo et al. 2012).

Studies have shown that noxious stimuli during pedicle traction and OVE increase systolic blood pressure and HR (Höglund et al. 2011, 2014). Tachycardia also occurs during laparoscopic surgery as a compensatory mechanism linked to the expansion of the peritoneal cavity and the stimulating effects of hypercapnia (Almeida et al. 2003). In our case, in line with other studies (Hancock et al. 2005; Höglund et al. 2011), no significant variations in HR were determined between the two surgical groups or between the four time points. This could probably be ascribed to the PNP values produced (8–9 mmHg), the type of premedication and anaesthesia, and a potential baroreceptor reflex resulting from elevated arterial pressure (Fernández‐Martín et al. 2022; Höglund et al. 2011).

In the research conducted by da Conceição et al., a rise in RR and EtCO_2_ was noted in the LapOVE group when compared to the mini COVE in felines. They linked this finding to the absorption of abdominal CO_2_, which reduces blood pH and increases RR (tachypnoea) (da Conceição et al. 2018). Moreover, previous studies have shown that significant respiratory alterations were observed during laparoscopy procedures, primarily linked to the rise in IAP, which resulted in the cranial displacement of the diaphragm, leading to a decrease in lung expansion and a reduction in functional residual capacity (Fernández‐Martín et al. 2022; Di Bella et al. 2018; van Goethem et al. 2003).

Despite the minimally invasive nature of laparoscopy, both procedures resulted in similar postoperative pain. A potential reason for this discrepancy could be the visceral pain induced by CO_2_ pneumoperitoneum, which may offset the benefits of smaller incisions (da Conceição et al. 2018; Papparella et al. 2014). Some studies show that LapOVH/OVE promotes decreased pain postoperatively compared with open midline OVH/OVE (Devitt et al. 2005; Culp et al. 2009; Hancock et al. 2005; Gauthier et al. 2015; Lee and Choi 2015; Jeong et al. 2024). The use of bipolar electrocoagulation and smaller incisions in laparoscopic surgery may explain such results (Devitt et al. 2005).

Severe trauma may induce immune reactions at the WBC level after surgery, which is marked by higher neutrophil counts and a reduction in lymphocyte numbers (Espadas‐González et al. 2023). In our research, WBC counts and adult neutrophils decreased in both groups at 2 h after surgery compared to preoperative values and returned to baseline values 24 h after surgery. This is probably due to haemodilution (Fernández‐Martín et al. 2022). However, this decline was significant only in the LapOVE group. The findings aligned with recent publications by Fernández‐Martín et al., which also identified a significant reduction in WBC and neutrophils compared to baseline following LapOVE in healthy dogs. The authors attributed the slightly higher leukocyte counts observed in the COVE group to a greater inflammatory and immune response, determined by a more invasive and painful procedure than laparoscopic surgery (Devitt et al. 2005; Fernández‐Martín et al. 2022). These results contradict previous studies that showed a significant increase after laparotomy and laparoscopic surgeries (Kumar et al. 2023; Dalmolin et al. 2024; Espadas‐González et al. 2023).

The lymphopenia and eosinopenia observed in the COVE group at 2 and 24 h after surgery, respectively, align with findings from the study on celiotomy and laparoscopic sterilization in dogs (Dalmolin et al. 2024). Dalmolin attributed this finding to the suspensory ligament rupture and ligature (suture) application (Dalmolin et al. 2024).

An imbalance between oxidants and antioxidants can result in oxidative stress, which is associated with hastened ageing and the onset of cardiovascular, skin and cancer‐related diseases across various species (Tomsič and Nemec Svete 2022; Dissemond et al. 2002; Nemec Svete et al. 2021; Burbaitė et al. 2024). The research demonstrated significant differences in oxidative stress indicators across the two groups. MDA levels were markedly elevated in the COVE group, indicating increased oxidative damage. The result aligns with prior studies suggesting that open procedures, marked by significant tissue manipulation and exposure, are linked to increased oxidative stress (Serin et al. 2008; Costa et al. 2024; Bruschetta et al. 2024; Yilmaz et al. 2014). Conversely, MDA levels in the LapOVE group were comparatively lower, highlighting the advantages of minimally invasive methods in diminishing lipid peroxidation despite the insufflation of CO_2_ gas into the abdominal cavity. The findings are consistent with Kumari et al. (2022) study, which demonstrated that midline laparoscopic OVE resulted in a significantly lower MDA level than COVE and laparoscopic triangle OVE. Nonetheless, in this research, the MDA index showed a notable increase 4 days post‐surgery across all groups involved in the study (Kumari et al. 2022). Certain findings indicate that levels of tissue oxygen tension and MDA rise following laparoscopic surgery. Additionally, numerous studies have demonstrated that OVH does not significantly impact serum MDA levels in dogs (Yilmaz et al. 2014). Other research has indicated that OVH in female dogs increases serum MDA levels on the third day post‐surgery (Lee 2012).

The TAC assay can track the antioxidant response by evaluating the levels of antioxidants in biological samples and their ability to counteract free radicals (Dalmolin et al. 2024; Tomsič and Nemec Svete 2022; Burbaitė et al. 2024; Lee 2012). TAS levels were significantly lower in the LapOVE group than in the COVE group during the 1‐week postoperative period. This may be due to the physiological effects of CO_2_ pneumoperitoneum used during laparoscopy, which can induce ischemia–reperfusion injury and possible organ ischaemia due to oxidative stress (Sammour et al. 2009; Nickkholgh et al. 2008). A previous study showed that plasma TAS levels significantly reduced after laparoscopic surgery (Glantzounis et al. 2001). The study by Lee et al. reported decreased plasma TAS levels in dogs when the pneumoperitoneum setting was performed at 15 mmHg rather than 12 mmHg or less. However, this was not statistically significant between groups (Lee and Choi 2015). Additionally, research has demonstrated in dogs that elevating the temperature of CO_2_ can lead to an increased inflammatory response and the production of ROS in both plasma and the peritoneal cavity (Milech et al. 2021). The results of this study and previous reports indicate that a diminished plasma TAS level may signify alterations in antioxidant status induced by pneumoperitoneum during anaesthesia (Lee and Choi 2015). These findings suggest using plasma antioxidants produced by ROS during the procedure (Lee and Choi 2015).

Several limitations in this study can be identified: (1) The sample size was relatively small, which reduces the statistical power to detect modest differences, particularly for subjective measures such as pain scores and for physiological parameters. Post‐hoc power analysis indicated very low power for pain outcomes (∼5%–20%) and physiological parameters (∼20%–25%), reflecting small effect sizes and limiting the ability to detect subtle between‐group differences. The sampling intervals were also relatively sparse, which may have missed short‐term or transient fluctuations. Future studies with larger cohorts and more frequent time points are warranted to validate and extend the present results. (2) The amount of blood loss was not recorded during either surgery, which could limit our study's findings. (3) Investigating cortisol levels and their connection with the analytes assessed here might provide additional insights into the effectiveness of these markers in assessing surgical stress. (4) Ultimately, TAS assesses the antioxidant capacity of serum but does not assess the function of key enzymes like catalases, glutathione peroxidase, and superoxide dismutase (Gautier et al. 2020). Measuring individual antioxidants together can offer a more comprehensive view of antioxidant status. (5) The results are not general; comparisons to other studies should be done cautiously. The haemodynamic response may be influenced by a variety of factors such as fluid balance and the drugs used for premedication and anaesthesia (Väisänen et al. 2002).

## 5 Conclusions

In conclusion, both surgical methods have demonstrated their safety and effectiveness for OVE in dogs. However, the COVE group exhibited significantly higher oxidative and inflammatory responses during the first week postoperatively, indicating greater surgical stress compared to the LapOVE group. Therefore, in veterinary practices where equipment is available, laparoscopy should be considered a safe option for performing OVE in dogs due to its lower invasiveness and reduced postoperative inflammation.

## Author Contributions

**Reza Naghavi**: conceptualization, methodology, validation, investigation. **Hossein Kazemi Mehrjerdi**: conceptualization, methodology, software, validation, investigation. **Mohammad Heidarpour**: conceptualization, methodology, software, validation, formal analysis, investigation.

## Funding

The authors have nothing to report.

## Disclosure

There was no financial support that could have influenced the study design, results, data analysis or writing.

## Conflicts of Interest

The authors declare no conflicts of interest.

## Data Availability

The data that support the findings of this study are available from the corresponding author upon reasonable request.
